# Integrated Multi-Omics Reveal the Genetic and Metabolic Blueprint for Corn Straw Degradation in the White-Rot Fungus *Irpex lacteus* J2

**DOI:** 10.3390/biology14101339

**Published:** 2025-10-01

**Authors:** Jian Pang, Shizhen Zhao, Tao Hua, Jiahui Fan, Zhe Yan, Mingyuan Chen, Fan Zhao, Jingshi Yu, Qiaoxia Shang

**Affiliations:** 1 Key Laboratory for Northern Urban Agriculture of Ministry of Agriculture and Rural Affairs, Beijing University of Agriculture, Beijing 102206, China; pangjian@bua.edu.cn (J.P.);; 2 Changping District Agricultural Service Center of Beijing, Beijing 102200, China; 3 Changping District Station for Popularizing Agricultural Technique of Beijing, Beijing 102299, China

**Keywords:** *Irpex lacteus* J2, whole-genome sequencing, corn straw, bioconversion, untargeted metabolomics

## Abstract

Lignocellulosic agricultural residues represent a rich source of potential feedstock for biorefinery applications, but their valorization remains challenging. The white-rot fungus *Irpex lacteus* J2 exhibited a promising degradation effect, but its molecular mechanisms of lignocellulose degradation remained largely uncharacterized. Here, we performed high-quality whole-genome sequencing and untargeted metabolomic profiling of *I. lacteus* J2 during the degradation of corn straw as the sole carbon source. The assembled *I. lacteus* J2 genome contained 14,647 protein-coding genes, revealing a rich genetic repertoire for biomass degradation and secondary metabolite synthesis. Comparative genomics showed high synteny (mean amino acid sequence identity 92.28%) with *I. lacteus* Irplac1. Untargeted metabolomic analysis unveiled a dynamic metabolic landscape during corn straw fermentation. Dominant metabolite classes included organic acids and derivatives (27.32%) and lipids and lipid-like molecules (25.40%), as well as heterocyclic compounds (20.41%). KEGG pathway-enrichment analysis highlighted significant activation of core metabolic pathways, with prominent enrichment in global metabolism (160 metabolites), amino acid metabolism (99 metabolites), carbohydrate metabolism (24 metabolites), and lipid metabolism (19 metabolites). Fermentation profiles at 3 and 15 days demonstrated substantial metabolic reprogramming, with up to 210 upregulated and 166 downregulated metabolites. Correlation analyses further revealed complex metabolic interdependencies and potential regulatory roles of key compounds. These integrated multi-omics insights significantly expand our understanding of the genetic basis and metabolic versatility, enabling *I. lacteus* J2 to efficiently utilize lignocellulose. Our findings position *I. lacteus* J2 as a robust model strain and provide a valuable foundation for developing advanced fungus-based strategies for sustainable bioprocessing and valorization of agricultural residues.

## Simple Summary

Agricultural waste, like corn straw, is an abundant resource that could be converted into valuable products such as biofuels, but its tough structure makes this challenging. A powerful wood-decaying fungus, *Irpex lacteus* J2, can efficiently break down this material, but the exact molecular processes it uses have been a mystery. Our study aimed to create a detailed blueprint of how this fungus works by mapping its entire set of genes and tracking the thousands of chemical compounds it produces while digesting corn straw. We discovered that the fungus possesses a rich genetic toolkit specifically for this task and that it dynamically alters its internal chemistry over time to effectively break down the plant matter. This research provided a fundamental understanding of the intricate strategies employed by this fungus to deconstruct agricultural waste. This knowledge was valuable because it could guide future efforts to enhance this process, paving the way for more efficient and sustainable methods to convert waste into renewable energy and other bio-based products.

## 1. Introduction

Lignocellulosic biomass such as corn straw is a highly abundant and inexpensive resource, but its valorization remains challenging [1]. Corn straw as an abundant agricultural residue holds significant promise as a sustainable feedstock for diverse biotechnological applications, including the production of biofuels, biochemicals, and enzymes. However, these residues are rich in cellulose, hemicellulose, and lignin, which confer recalcitrance to biodegradation and limit their spontaneous decomposition in the environment [2]. Efficient and sustainable strategies for valorizing these materials into high-value products are urgently needed to support global goals in waste management, bioenergy, and circular bioeconomy development [3].

Among various bioconversion strategies, microbial degradation has emerged as a promising and environmentally friendly alternative to traditional chemical or physical pretreatment methods [4]. Microorganisms, particularly wood-decay fungi, play a central role in natural biomass turnover due to their ability to secrete a broad range of lignocellulolytic enzymes [5]. These fungi can decompose complex plant polymers and convert them into valuable metabolites, including lipids, organic acids, and bioactive compounds. Their metabolic versatility and adaptive capabilities make them suitable candidates for the bioconversion of renewable biomass into industrially relevant compounds.

*Irpex lacteus* (Fr.) Fr. (Phanerochaetaceae) is a basidiomycetous white-rot fungus that usually colonizes deadwood [6], with notable lignocellulolytic activity and stress tolerance [7]. As a result, this fungus has garnered increasing attention for its potential in lignocellulose degradation and waste valorization. Despite its promising characteristics, the molecular mechanisms underlying its metabolic plasticity and biomass-degrading capabilities remain poorly characterized. Few studies to date have systematically explored the genomic architecture or metabolic network of *I. lacteus*, especially when grown on lignocellulose. For example, Zhu et al. examined the effects of sterilization methods on the degradation ability and bioactive compound production of *I. lacteus* grown on wheat straw through metabolomic analysis [8]. Wang et al. analyzed the genome sequence and metabolic potential of *I. lacteus* Y1 using a PacBio Sequel II sequencer, with the aim of facilitating further drug research and functional food production [9]. While some preliminary omics studies have been reported, a comprehensive understanding of functional genes, pathway regulation, and substrate-specific metabolic responses is still lacking. The integration of high-resolution whole-genome sequencing with metabolomic profiling represents a powerful approach to uncover the fundamental biological processes driving substrate utilization and metabolite production.

Fungal genomes are typically large and complex, so traditional sequencing technologies generally cannot meet the demands of data-intensive comprehensive fungal genomic research. However, the rapid advancement of next-generation high-throughput sequencing (NGS) and third-generation single-molecule real-time (SMRT) sequencing technologies characterized by high throughput, high accuracy, ultra-long read lengths, and fast turnaround has greatly simplified de novo genome assembly in fungi. These technologies enable the accurate prediction of key genes and proteins, thereby facilitating functional annotation and the elucidation of potential molecular mechanisms. As a next-generation omics technology that emerged after genomics, transcriptomics, and proteomics, metabolomics represents a crucial tool of systems biology. It focuses on dynamic changes of metabolite levels within biological systems over time or in response to environmental perturbations. By identifying differential metabolites and mapping them to associated biological pathways, metabolomics provides valuable insights into the molecular mechanisms underlying physiological and pathological processes.

In this study, we generated a high-quality whole-genome sequence of *I. lacteus* J2 and performed untargeted metabolomic analysis of mycelia grown on corn straw as the sole carbon source. Based on these large datasets, we comprehensively characterized the genomic features and functional gene categories associated with biomass degradation and secondary metabolism. Further metabolomic analysis identified key pathways and metabolites that were upregulated during lignocellulosic substrate utilization. The results of this study expand our understanding of *I. lacteus* J2 as a model strain for biomass valorization and contribute to the development of fungus-based strategies for sustainable bioprocessing of agricultural residues.

## 2. Materials and Methods

### 2.1. Strain and Culture Conditions

*I. lacteus* J2 was isolated from old trees in The Summer Palace (Beijing, China) and has been deposited in the China Center for Type Culture Collection (CCTCC) under accession number M20232318. The preserved fungal strains were first reactivated on potato dextrose agar (PDA). PDA medium (g/L): peeled potato-200.0, glucose-20.0, and agar-20.0 with a natural pH of 5.8. Vigorous and healthy mycelia were then selected and transferred into liquid medium to prepare the seed culture, which was used to inoculate fermentation medium at an inoculation rate of 5% (*v*/*v*) and then incubated at 28 °C for 15 days. Liquid medium: 200 g/L potato extract, 20 g/L glucose with a natural pH of 5.8. Fermentation medium (g/L): corn straw-10.0, (NH_4_)_2_C_4_H_4_O_6_-0.2, KH_2_PO_4_-2.0, MgSO_4_·7H_2_O-0.71, CaCl_2_-0.1 and 70 mL of microelement solution (g/L): NaCl-1.0, CoCl_2_·6H_2_O-0.184, FeSO_4_·7H_2_O-0.1, ZnSO_4_·7H_2_O-0.1, CuSO_4_-0.1, H_3_BO_3_-0.01, Na_2_MoO_4_·2H_2_O-0.01, KAl (SO_4_)_2_·12H_2_O-0.01, and C_6_H_9_NO_6_-1.5. All the chemicals used in this study were of molecular biology or analytical grade (Sinopharm Chemical Reagent Co., Ltd., Shanghai, China). Corn straw (pioneer) was obtained from Dandong (Liaoning, China).

### 2.2. Genome Extraction, Sequencing, and Assembly

*I. lacteus* J2 was grown on PDA plates for 5 days at 28 °C, after which the genomic DNA was extracted using a commercial extraction kit (Tiangen Biochemical Technology Co., Ltd., Beijing, China). The whole genome of *I. lacteus* J2 was sequenced using the DNBSEQ-G400/T7/T10 and PacBio sequencing platform. For second-generation DNBSEQ sequencing, the genomic DNA was randomly fragmented by Covaris, then short DNA fragments of the desired length were obtained by adjusting the fragmentation parameters [10]. The fragmented samples were size-selected using an Agencourt AMPure XP-Medium kit to concentrate the DNA fragments around 300–400 bp. A reaction mixture was prepared, and the reaction was allowed to proceed for a specific time at the appropriate temperature to repair the ends of the double-stranded DNA and add an A base to the 3′ ends. A ligation reaction mixture was then prepared, and the reaction was allowed to proceed for a specific time at the appropriate temperature to ligate the adaptors to the DNA. A PCR reaction mixture was prepared to amplify the ligation products. After denaturing the PCR products into single-stranded DNA, a circularization reaction mixture was prepared. The reaction yielded single-stranded circular products. Following digestion of uncircularized linear DNA molecules, the final library was obtained. Single-stranded circular DNA molecules undergo rolling circle replication to form DNA nanoballs (DNBs), each containing over 300 copies. The resulting DNBs are then applied to the patterned wells on a high-density DNA nanochip. Sequencing is performed using combinatorial probe-anchor synthesis (cPAS) technology. For third-generation PacBio sequencing, the starting sample is the extracted genomic DNA, which is sheared (about 10–15 K), digested by enzyme reaction, put through damage repair and terminal repair, and connected with the barcode sequence linker and BluePippin sorting. Finally, a SMRTbell library is finally obtained. The qualified library will be sequenced on the PacBio platform. The raw data of PacBio RS II sequencing were saved in H5 format, which could not be edited or viewed directly and needed to be converted into fasta format [11,12]. However, the subreads contained 15% indel errors, so in each ZMW, a consistent sequence analysis was needed to obtain more precise Circular Consensus Sequencing Data (CCS, also called Reads of Insert). CCS would be used directly for assembly, alignment, and species classification. We removed the adapter from the polymerase reads and thus obtained several subreads, each of which was converted into a CCS after processing. Subreads shorter than 1000 bp were removed, and the high-quality reads were assembled as described before [12,13,14,15]. The PacBio platform generated a certain amount of low-quality data and adapter sequences in polymerase reads. In order to obtain more accurate and reliable results for subsequent bioinformatic analysis, the raw data were filtered to remove the low-quality data.

### 2.3. Gene Function Analysis

Functional annotation was based on an analysis of protein sequences. To ensure biological meaning, the highest-quality alignment result was chosen as gene annotation. Functional annotation was completed by Diamond software (v0.8.23.85) against the GO (Gene Ontology) [16,17], KEGG (Kyoto Encyclopedia of Genes and Genomes) [18], COG (Clusters of Orthologous Groups) [19], NR (Non-Redundant Protein Database databases) [20], TCDB (Transporter Classification Database) [21], and Swiss-Prot databases [22]. A whole-genome BLAST search (2.13.0) (E-value of less than 1 × 10^−5^, minimal alignment length above 40%) was performed against the seven indicated databases. In addition, gene clusters related to secondary metabolites were analyzed using antiSMASH, and genes encoding carbohydrate-active enzymes were predicted using the Carbohydrate-Active enZYmes (CAZY) Database [23].

### 2.4. Comparative Genomics Analysis

The genome size, number of contigs, contig/scaffold N50, contig/scaffold L50, and GC content of *I. lacteus* Irplac1 were 47.7 Mb, 424, 476.3 kb, 24, and 50.5%, respectively. Genomic alignment between *I. lacteus* J2 and a published Irplac1 genome (GCA_022606095.1) was performed using the MUMmer and LASTZ tools [24]. The sequence of the target is ordered according to that of the reference sequence based on Mummer. Then, the upper and following axes of linear synteny graph are constructed after the same proportion of size reduction in length of both sequences. The protein set P1 of target sequence is aligned with the protein set P2 of reference sequence. Firstly, P1 is aligned with P2 in BLASTp (2.13.0) m8 by taking P2 as database, e-value ≤ 1 × 10^−5^, and identity ≥, and the best hit of each protein is selected; secondly, the same alignment is carried out by taking P1 as database; finally the results with best hit value for both alignments are reserved and the consistent value is the average of two consistent values. Each pair of best hits for two alignments is marked in the coordinate diagram according to its position information after the same proportion of size reduction.

### 2.5. Metabolomic Profiling of I. lacteus J2 During Corn Straw Bioconversion

*I. lacteus* J2 was grown in 50 mL of fermentation medium in a 300 mL flask at 28 °C with shaking at 180 rpm for 15 days. To investigate the temporal dynamics of metabolites during the fermentation of corn straw by *I. lacteus* J2, samples were collected on days 3, 9, and 15 for differential metabolite analysis. On days 3, 9, and 15 of *I. lacteus* J2 cultivation, fermentation broth was sampled. Three independent biological replicates were taken at each time point. The collected fermentation broth was then centrifuged at 15,000× *g* for 10 min at 4 °C, after which the supernatant was collected and the precipitate was discarded. Subsequently, samples (1 mL) were collected on days 3, 9, and 15, freeze-dried, mixed well with prechilled 80% methanol by vortexing, and incubated on ice for 5 min. After centrifugation at 15,000× *g* and 4 °C for 15 min, the supernatant was diluted to a final concentration of 53% methanol with ultrapure water and transferred to a fresh EP tube. After another centrifugation at 15,000× *g* and 4 °C for 15 min, the supernatant was used as the Liquid Chromatography—Tandem Mass Spectrometry (LC-MS/MS) sample.

### 2.6. Ultra-High-Performance Liquid Chromatography—Tandem Mass Spectrometry (UHPLC-MS/MS) Analysis of Metabolites Produced by I. lacteus J2 from Corn Straw

This study used a Vanquish UHPLC system coupled with an Orbitrap Q ExactiveTM HF or HF-X mass spectrometer (Thermo Fisher Scientific Inc., Waltham, Massachusetts, USA) at Novogene Co., Ltd. (Beijing, China). The Hypersil Gold Column (100 × 2.1 mm, 1.9 μm) was eluted via a 12 min linear gradient at a flow rate of 0.2 mL/min, comprising eluent A (0.1% FA in water) and eluent B (methanol). The solvent gradient was set as follows: 2% B, 1.5 min; 2–85% B, 3 min; 85–100% B, 10 min; 100–2% B, 10.1 min; 2% B, 12 min. The Q ExactiveTM HF mass spectrometer was operated in positive/negative ion mode with spray voltage of 3.5 kV, capillary temperature of 320 °C, Aux gas heater temperature of 350 °C, aux gas flow rate of 10 L/min, sheath gas flow rate of 35 psi, and S-lens RF level of 60.

### 2.7. Data Processing and Metabolite Identification

To perform peak alignment, picking, and quantitation for each metabolite, the UHPLC-MS/MS data files were processed using Extracted Chromatographic Mass Spectrometry (XCMS). Based on adductions and a mass deviation setting of 10 ppm, a comparison was made between these data and the high-quality secondary spectrum database to obtain results for metabolite identification. After eliminating background ions based on blank samples, the original quantitative results were normalized to obtain relative peak areas using the formula: Relative peak areas = Raw quantitative value of samples/(The sum of quantitative value of samples/The sum of quantitative value of Quality Control 1, QC1); compounds with a coefficient of variation (CV) of relative peak areas in QC samples greater than 30% were removed. Finally, the identification and relative quantification results of metabolites were obtained. Data processing was conducted using R (3.4.3) and Python (3.5.0) in the Linux operating system (CentOS version 6.6). For details on specific packages and software versions used, please refer to the README file included in the results.

### 2.8. Data Analysis

Metabolite annotation was based on the HMDB: https://hmdb.ca/metabolites (accessed on 1 March 2025), KEGG: https://www.genome.jp/kegg/pathway.html (accessed on 1 March 2025), and LIPIDMaps: http://www.lipidmaps.org/ (accessed on 1 March 2025) databases. Partial least squares discriminant analysis (PLS-DA) and principal component analysis (PCA) were conducted in metaX [25]. Univariate analysis (*t*-test) was used to assess the statistical significance of differences. Differential metabolites were selected based on a *p*-value < 0.05, (Variable Importance in the Projection, VIP) > 1, and fold change ≥ 2 or FC ≤ 0.5. Metabolites of interest were filtered via volcano plots based on log2 (Fold Change) and -log10 (*p*-value) of metabolites using the R package (3.4.3) ggplot2. Clustering heatmaps were plotted using the R package Pheatmap (3.4.3) using data that were normalized based on z-scores of the intensity areas of differential metabolites. The functions of the identified differential metabolites and metabolic pathways were studied using the KEGG database.

## 3. Results

### 3.1. Genome Sequence Assembly and Annotation

The PacBio platform generated a certain amount of low-quality data and adapter sequences in polymerase reads. In order to obtain more accurate and reliable results for subsequent bioinformatic analysis, the raw data were filtered to remove the low-quality data. As shown in Table 1, the number of valid Zero-Mode Waveguides (ZWMs) and subreads was 127,431 and 1,546,034, respectively. The subreads of total bases, mean length, N50, N90, max length, and min length were 15,016,142,153 bp, 9712 bp, 10,169 bp, 6630 bp, 245,281 bp, and 2000 bp, respectively. Next, the clean data were assembled for each sample, which was followed by multiple adjustments to achieve the optimal assembly results with 114 contigs. The genome size of *I. lacteus* J2 was 46,386,314 bp, with a GC content of 50.22%. The N50 value was 2,420,162 bp, the N90 value was 297,826 bp, the longest contig length was 4,106,595 bp, and the shortest contig length was 9353 bp. The genome was predicted to contain a total of 14,647 protein-coding genes, with an average coding sequence length of 1548 bp. The total linkage length was 22,675,219 bp, accounting for 48.88% of the genome sequence. Among non-coding RNAs, the genome of *I. lacteus* J2 was predicted to encode 295 transfer RNAs (tRNAs), 33 ribosomal RNAs (rRNAs), 45 small nuclear RNAs (snRNAs), and 98 microRNAs (miRNAs).

**Figure 1:**
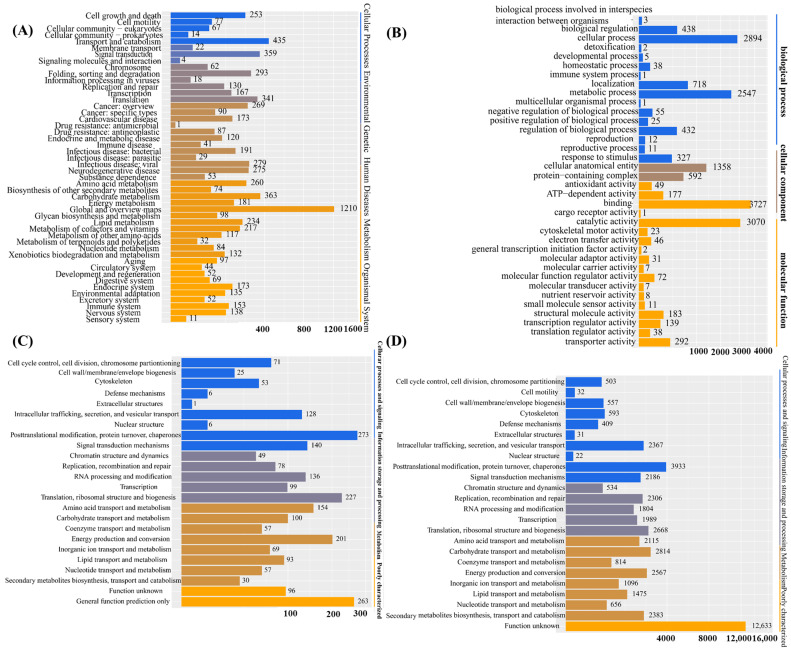
Functional annotation based on the KEGG, GO, KOG, and NOG databases. (**A**) The Kyoto Encyclopedia of Genes and Genomes (KEGG) database divides biological pathways into eight main categories, each of which comprises several subcategories; (**B**) The Gene Ontology (GO) database is divided into three main parts: cellular component, molecular function, and biological process; (**C**) KOG is a protein database created and maintained by NCBI. The database is based on the evolutionary relationships of protein systems among bacteria, algae, and eukaryotes. (**D**) Non-supervised Orthologous Groups (NOG) database. Annotations for functional description and functional classification of direct homologous groups.

Upon obtaining the gene set of *I. lacteus* J2, functional annotation through database searches was conducted. This process enabled a comprehensive functional classification of the genomic content and facilitated the identification of target genes for downstream research. A total of 13,492 protein-coding genes were annotated based on the KEGG (4730/32.29%), GO (6882/46.98%), NOG (7469/50.99%), and KOG (2169/14.8%) databases. KEGG pathway-enrichment analysis demonstrated a prominent enrichment of genes involved in metabolic and signaling pathways. The most representative metabolic category was global and overview maps, comprising 1210 genes, followed by carbohydrate metabolism (363 genes), lipid metabolism (234 genes), and energy metabolism (181 genes). Additional key metabolic pathways included amino acid metabolism (260 genes), metabolism of cofactors and vitamins (217 genes), and glycan biosynthesis and metabolism (98 genes), highlighting the broad involvement of the annotated genes in core biochemical processes. In terms of signaling pathways, significant gene enrichment was observed in signal transduction (359 genes), signaling molecules and interactions (62 genes), and membrane transport (22 genes). These results suggested an active role of the analyzed genes in intercellular communication and intracellular signaling cascades. Furthermore, genes associated with replication and repair (293 genes), as well as protein folding, sorting, and degradation (130 genes), suggested a close connection between metabolic activities and cellular quality-control mechanisms. Overall, these findings indicated that the identified genes were extensively involved in essential metabolic functions and signal transduction processes, reflecting their central roles in maintaining cellular homeostasis (Figure 1A). As shown in Figure 1B, Gene Ontology (GO) annotation of the predicted genes of *I. lacteus* J2 revealed functional enrichment across the three main categories, biological process, cellular component, and molecular function, encompassing a total of 8958 genes. In the biological process category, the most prominent terms were cellular process (2894 genes), metabolic process (2547 genes), and localization (718 genes), reflecting active cellular dynamics and metabolic adaptability. Regulatory functions were also well represented, including biological regulation (438 genes) and regulation of biological processes (432 genes). In the cellular component category, cellular anatomical entity (1358 genes) and protein-containing complex (592 genes) were the most prominent, indicating extensive involvement in structural and macromolecular assemblies. Within the molecular function domain, catalytic activity was highly enriched (3070 genes), consistent with the organism’s lignocellulolytic capabilities. Substantial numbers of genes were also associated with binding (372 genes), transporter activity (292 genes), and ATP-dependent activity (177 genes), underscoring active transport and energy metabolism. The presence of genes related to antioxidant activity (49 genes) and electron transfer activity (46 genes) suggests roles in redox homeostasis and the oxidative stress response. Overall, the GO classification reflected the versatile genetic repertoire of *I. lacteus* J2, supporting its potential for biomass degradation and industrial bioconversion. Developed and maintained by the NCBI, the KOG database classifies proteins from complete genomes of bacteria, algae, and eukaryotes based on their evolutionary relationships (Figure 1C). Based on the KOG database, a total of 7469 genes were identified. According to the functional classification, categories with more than 200 genes included posttranslational modification, protein turnover, chaperones (273), translation, ribosomal structure, and biogenesis (227), as well as energy production and conversion (201). Additionally, categories with over 100 associated genes included intracellular trafficking, secretion, and vesicular transport (128), signal transduction mechanisms (140), RNA processing and modification (136), amino acid transport and metabolism (154), and carbohydrate transport and metabolism (100). NOG annotation of *I. lacteus* J2 revealed a wide functional spectrum. The largest group comprised genes with unknown function (12,633), indicating substantial uncharacterized genomic content. Among annotated categories, metabolic functions were prominent. The large numbers of genes involved in carbohydrate metabolism (2814), energy production (2567), amino acid metabolism (2115), and secondary metabolite biosynthesis (2383) suggested strong metabolic versatility. In the information storage and processing category, notable enrichment was observed in posttranslational modification (3933), translation (2668), and DNA replication and repair (2306), reflecting active cellular maintenance and protein turnover. Cellular processes and signaling were also well represented, particularly intracellular trafficking (2367), signal transduction (2186), and cytoskeleton-related functions (593), indicating a complex regulatory and transport system. Additionally, a large number of enzyme systems responsible for carbohydrate degradation, modification, and biosynthesis were identified through Carbohydrate-Active enZYmes (CAZy) database annotation, encompassing glycoside hydrolases, glycosyltransferases, polysaccharide lyases, carbohydrate esterases, and auxiliary activities. A comprehensive analysis of the *I. lacteus* J2 whole-genome data was also performed, which revealed the presence of several key lignocellulose degradation-related genes (Table 2).

### 3.2. Comparative Genomics Between I. lacteus J2 and Irplac1

**Figure 2:**
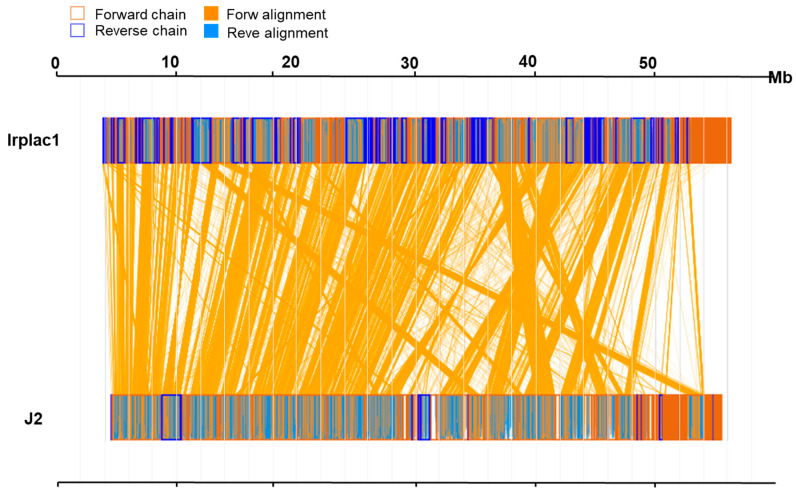
Genomic synteny analysis of *I. lacteus* J2 and Irplac1at the animo acid sequence level. In sequence box, the yellow region stands for the animo acid sequence in the forward chain of this genome sequence, and the blue region stands for the animo acid sequence in the reverse chain of this genome sequence. In the middle region of two sequences, the yellow line stands for forward alignment, and the blue line stands for reverse complementary alignment.

To investigate the whole-genome similarity between *I. lacteus* J2 and Irplac1, we performed a synteny analysis based on amino acid sequences. The results revealed a high degree of conserved collinearity, as evidenced by extensive forward alignment blocks (orange lines) between the two genomes (Figure 2). A total of 9708 genes were identified as collinear, representing 66.28% of the 14,647 genes in the genome of *I. lacteus* J2, as well as 63.38% of the 15,318 genes in the genome of Irplac1. The mean amino acid sequence identity of the aligned genes was 92.28%, with a median amino acid sequence identity of 96.24%, indicating that most orthologous gene pairs were highly conserved at the amino acid level. Notably, the alignment was predominantly in the forward direction, suggesting minimal large-scale structural rearrangements. Taken together, these results supported a close evolutionary relationship between *I. lacteus* J2 and Irplac1, providing a foundation for further functional and comparative genomic analyses.

### 3.3. Metabolomic Analysis of I. lacteus J2 During Corn Straw Degradation

**Figure 3:**
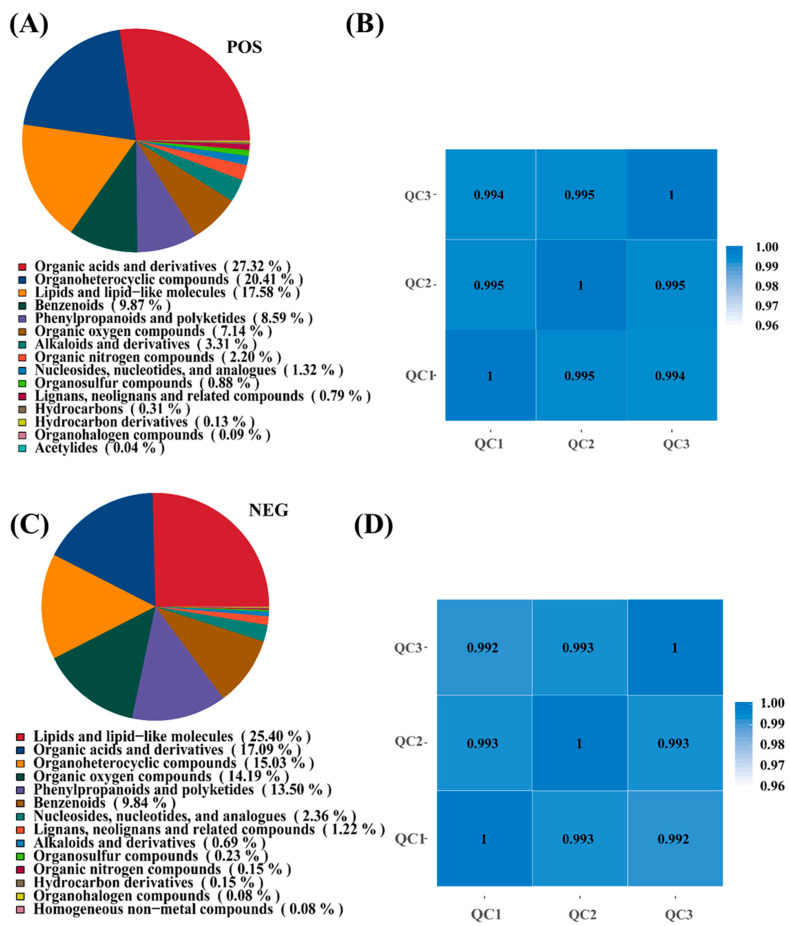
Metabolite pie chart and sample correlation analysis of corn straw fermentation by *I. lacteus* J2. (**A**,**B**): positive ion mode (POS); (**C**,**D**): negative ion mode (NEG). Quality Control (QC): Based on the relative quantitative values of metabolites, the Pearson correlation coefficient between QC samples is calculated. The higher the correlation of QC samples (the closer |r| is to 1), the better the stability of the entire detection process and the higher the data quality. QC samples inserted during sample detection are used to evaluate the stability of the system throughout the experimental process and to perform data quality control analysis.

To elucidate the metabolic mechanisms underlying *I. lacteus* J2-mediated corn straw degradation, untargeted metabolomic analysis was performed using both positive (POS) and negative (NEG) ionization modes. In the POS mode (Figure 3A), the dominant metabolite classes were organic acids and derivatives (27.32%), followed by heterocyclic compounds (20.41%), as well as lipids and lipid-like molecules (17.58%). Other notable categories included benzenoids (9.87%), phenylpropanoids, and polyketides (8.59%), as well as oxygen-containing organic compounds (7.14%). Minor classes such as alkaloids and derivatives (3.31%), as well as organic nitrogen compounds (2.20%), were also detected, reflecting the metabolic complexity of lignocellulose degradation. In NEG mode (Figure 3C), the metabolite profile showed a slight shift of dominant classes. Lipids and lipid-like molecules were most abundant (25.40%), followed by organic acids and derivatives (17.90%), as well as heterocyclic compounds (15.03%). Oxygen-containing organic compounds (14.19%), as well as phenylpropanoids and polyketides (13.50%), also accounted for significant proportions, whereas other classes, such as nucleosides, nucleotides, and analogues (2.36%), or alkaloids and derivatives (0.69%), were less represented. These results suggested that *I. lacteus* J2 produced a broad spectrum of metabolites during the degradation process, with prominent roles played by organic acids, lipids, and aromatic compounds. The differences between ion modes reflected complementary detection capacities, reinforcing the importance of dual-mode analysis in capturing the full metabolic landscape. High Pearson correlation coefficients (|r| ≈ 1), among QC samples in positive and negative ion mode, indicated excellent stability and data quality throughout the detection process. This strong correlation validated the high repeatability and reliability of the experimental results, ensuring the integrity and consistency of the dataset for subsequent analysis (Figure 3B,D).

### 3.4. KEGG Pathway and LIPID MAPS Enrichment Analysis

**Figure 4:**
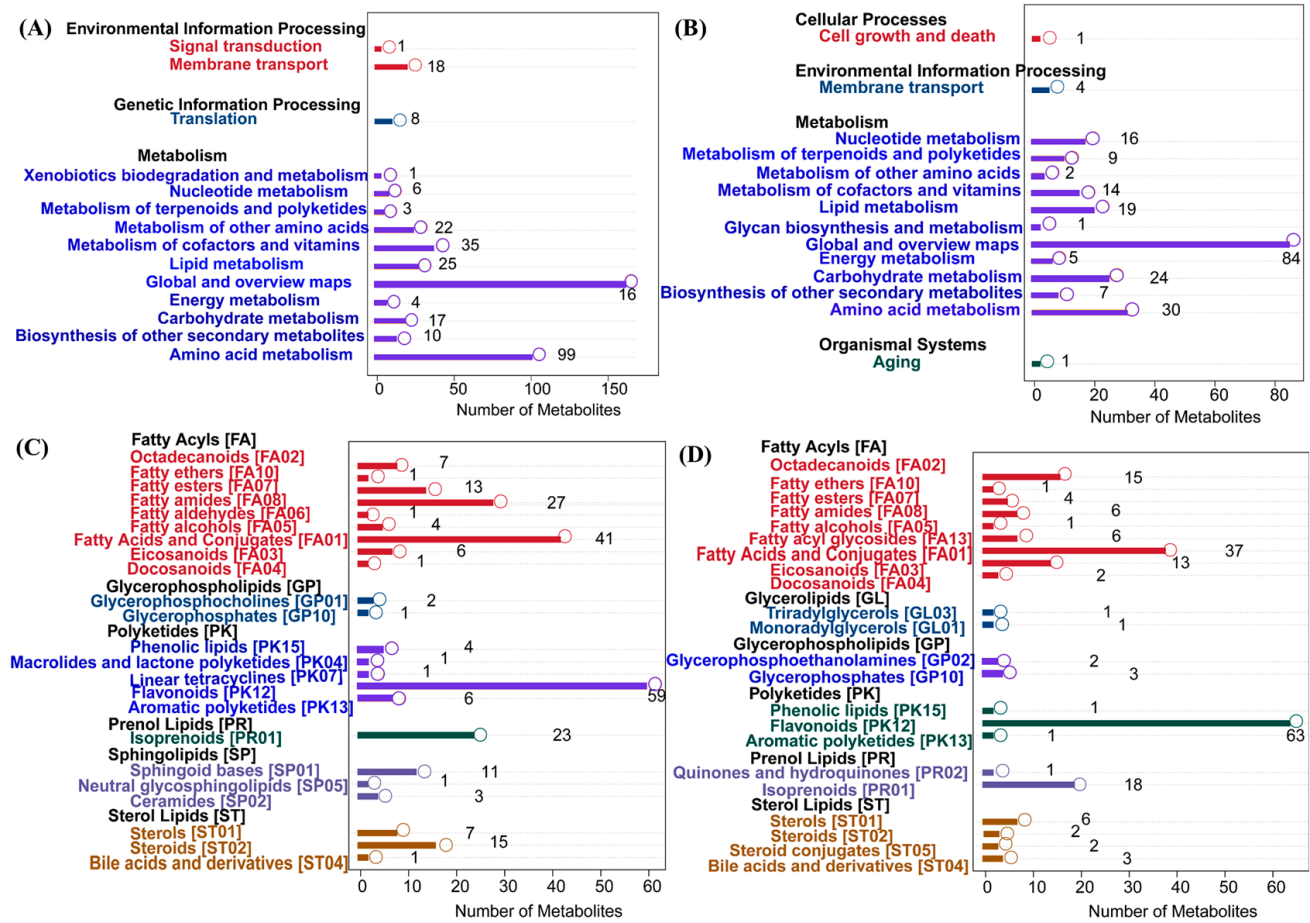
KEGG pathway annotation and LIPID MAPS classification. (**A**,**C**): positive ion mode; (**B**,**D**): negative ion mode.

In Figure 4, panels A and B illustrate the KEGG pathway-enrichment results of metabolites identified in positive and negative ion mode, respectively. A broader metabolic coverage was observed in positive ion mode, with significant enrichment in pathways including global and overview maps (160 metabolites) and amino acid metabolism (99), as well as the metabolism of cofactors and vitamins (35), indicating strong representation of core and vitamin-associated metabolic pathways. By contrast, the negative ion mode (panel B) revealed focused enrichment in amino acid metabolism (30 metabolites), carbohydrate metabolism (24), and lipid metabolism (19), with representative pathways such as glycan biosynthesis and metabolism, as well as the biosynthesis of other secondary metabolites. These results suggested that the positive ion mode was more sensitive to negatively charged metabolites and complex glycans. Together, the two ion modes offered complementary metabolic insights, with the positive ion mode favoring detection of core metabolic intermediates and cofactors, while the negative ion mode offered enhanced coverage of sugar derivatives and secondary metabolites. This dual-mode strategy enabled a more comprehensive characterization of the metabolic landscape. Panels C and D illustrate the classification of lipids into eight major categories and their subclasses based on the LIPID MAPS database in positive and negative ion modes, respectively. In positive ion mode (panel C), lipids were primarily annotated as fatty acyls, glycerolipids, and glycerophospholipids, with a notable abundance of glycerophosphocholines and triacylglycerols. In negative ion mode (panel D), lipids showed a broader distribution, with particular enrichment in acidic subclasses such as glycerophosphoethanolamines, fatty acids, and conjugates, as well as polyketides, including flavonoids and phenolic lipids. Overall, the combined use of positive and negative ion modes provided complementary coverage, enabling a more comprehensive and detailed lipidome characterization.

### 3.5. Functional Analysis of Differential Metabolites

**Figure 5:**
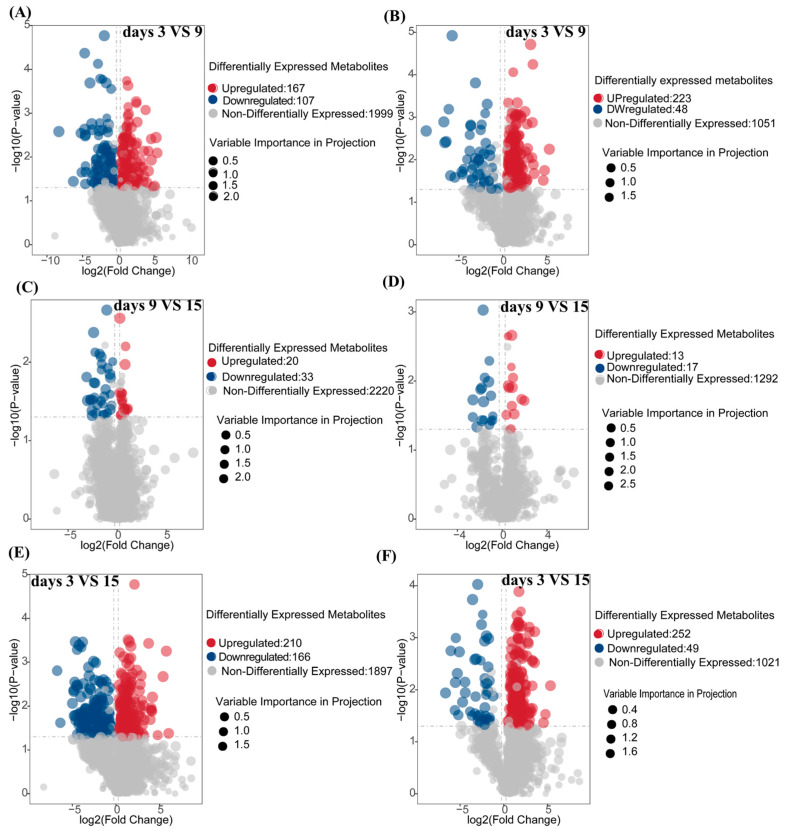
Volcano plot of differential metabolites in positive and negative ion mode during fermentation on days 3, 9, and 15. (**A**,**C**,**E**): positive ion mode; (**B**,**D**,**F**): negative ion mode. (**A**,**B**): Analysis of the number of differential metabolites between fermentation days 3 and 9; (**C**,**D**): Analysis of the number of differential metabolites between fermentation days 9 and 15 (**E**,**F**): Analysis of the number of differential metabolites between fermentation days 3 and 15. The *p*-value was calculated using a T-test, indicating the level of statistical significance.

To investigate the temporal dynamics of metabolites during the fermentation of corn straw by *I. lacteus* J2, samples were collected on days 3, 9, and 15 for differential metabolite analysis. The volcano plots in panels A–F illustrate the overall distribution of differentially expressed metabolites in both positive (POS) and negative (NEG) ion modes for various comparisons: fermentation day 9 vs. day 3 (A, B), fermentation day 15 vs. 9 (C, D), and fermentation day 15 vs. 3 (E, F). In positive ion mode (A, C, E), significant metabolites were clearly separated, with the majority of the upregulated (red) and downregulated (blue) metabolites exhibiting marked shifts of fold-change and corresponding *p*-values. Figure 5A (9d vs. 3d) showed 167 upregulated and 107 downregulated metabolites, with a prominent spread of data points at higher *p*-values and fold-changes. In the 15d vs. 9d comparison (Figure 5C), 20 metabolites were upregulated and 33 were downregulated, while the overall number of non-differential metabolites was considerably higher (2220). Figure 5E (15d vs. 3d) displayed the largest number of significant changes, with 210 upregulated and 166 downregulated metabolites. Similar trends were observed in negative ion mode (B, D, F), albeit with slight variations in the distribution. Figure 5B (9d vs. 3d) showed a greater number of upregulated metabolites (223) compared to downregulated ones (48), with a similar distribution pattern for non-differential metabolites (1051). In the 15d vs. 9d comparison (Figure 5D), 13 metabolites were upregulated and 17 downregulated. The most notable distribution was seen in Figure 5F (15d vs. 3d), where a significant shift in upregulated metabolites (252) was observed compared to downregulated ones (49), aligning with the trends seen in positive ion mode.

**Figure 6:**
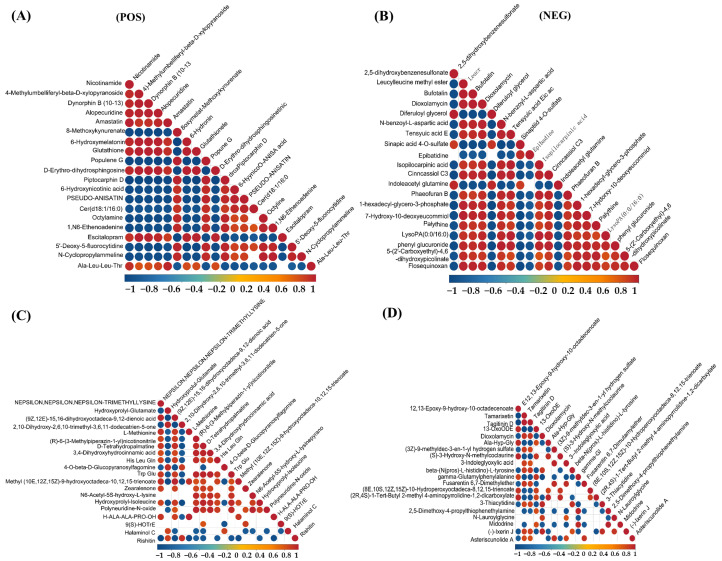

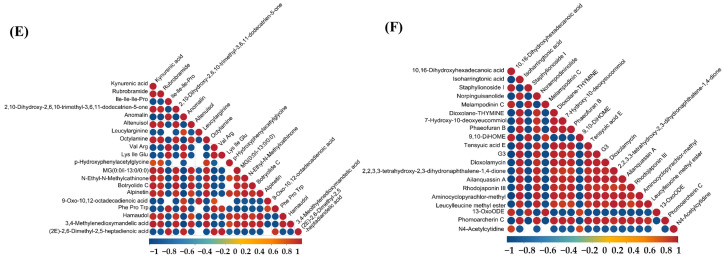
Differential metabolite correlation analysis of corn straw degradation by *I. lacteus* J2. (**A**,**C**,**E**): positive ion mode; (**B**,**D**,**F**): negative ion mode. (**A**,**B**): Differential metabolite correlation between fermentation days 9 and 3; (**C**,**D**): Differential metabolite correlation between fermentation days 15 and 9; (**E**,**F**): Differential metabolite correlation between fermentation days 15 and 3.

As shown in Figure 6, the correlation analysis of differential metabolites in the corn-straw-based fermentation broth of *I. lacteus* J2 was illustrated in both positive (POS) and negative (NEG) ion modes. The correlations were calculated using Pearson’s correlation coefficients between pairs of metabolites to assess the consistency of metabolic trends over time. Significant correlations were determined using *p*-value < 0.05 as the threshold, indicating a significant relationship between metabolites. In positive ion mode, a distinct cluster of red circles near the diagonal showed that certain metabolites, such as nicotinamide, 4-methylumbelliferyl-β-D-xylopyranoside, Dynorphin B (10–13), Alopecuridine, and Amastatin exhibited strong positive correlations, suggesting that they likely shared common metabolic pathways or were co-regulated during the fermentation process. However, Nicotinamide and Piptocarphin D, 6-Hydroxynicotinic acid and Pseudo-anisatin exhibited strong negative correlations with other metabolites (Figure 6A). By contrast, strong positive correlations were observed between Nε,Nε,Nε-trimethyllysine and (9Z,12E)-15,16-dihydroxyoctadeca-9,12-dienoic acid, L-Methionine, (R)-6-(3-Methylpiperazin-1-yl)nicotinonitrile, D-Tetrahydropalmatine, and 3,4-Dihydroxyhydrocinnamic acid. Conversely, rishitin exhibited strong negative correlation with several metabolites, including Nε,Nε,Nε-trimethyllysine and hydroxyprolyl-glutamate, suggesting a potentially divergent role (Figure 6C). As shown in Figure 6E, strong positive correlations were observed among several amino acids and related compounds, including kynurenic acid, Rubrobramide, and Altenuisol, indicating coordinated metabolic activity. Kynurenic acid, Rubrobramide, Altenuisol and Ile-Ile-Ile-Pro, and Anomalin showed a strong negative correlation, suggesting divergent metabolic pathways. In negative ion mode, metabolites such as 2,5-dihydroxybenzenesulfonate and Leucylleucine methyl ester showed positive correlations with each other, shared metabolic precursors. However, the other 18 compounds displayed negative correlations with 2,5-dihydroxybenzenesulfonate and Leucylleucine methyl ester, suggesting diverse metabolic shifts (Figure 6B). As shown in Figure 6D, 12,13-epoxy-9-hydroxy-10-octadecenoate and Tamarixetin, Tagitinin D, and 13-OxoODE showed a strong positive correlation (r > 0.8), potentially indicating coordinated regulation or shared biosynthetic pathways. The four compounds in this group showed a very strong negative correlation with the majority of the other metabolites. In Figure 6F, 10,16-Dihydroxyhexadecanoic acid and Staphylionoside I, Melampodinin C, Dioxolane-Thymine showed a very strong positive correlation with each other. This group showed a very strong negative correlation with other metabolites, such as Isoharringtonic acid, Norpinguisanolide, 7-Hydroxy-10-deoxyeucommiol, and Phaeofuran B.

## 4. Discussion

Lignocellulosic biomass represents a great untapped resource for sustainable bioconversion technologies. White-rot fungi, such as *I. lacteus*, are promising hosts for biomass valorization due to their potent lignocellulolytic machinery [26]. However, the underlying molecular mechanisms of lignocellulose degradation by this fungus remained largely uncharacterized. This study addressed this gap by employing an integrated multi-omics approach, combining high-quality whole-genome sequencing with untargeted metabolomics to elucidate the molecular strategies employed by *I. lacteus* J2 during corn straw degradation.

Genomic analysis of *I. lacteus* J2 revealed a robust genetic architecture comprising 14,647 protein-coding genes. This extensive repertoire, notably its rich complement of CAZymes and oxidoreductases, provides the foundational enzymatic toolkit for efficient plant cell wall degradation [23]. Specifically, the identification of diverse CAZy families directly underpinned the fungus’s remarkable capacity to dismantle complex lignocellulose into simpler sugars and aromatic compounds, which was subsequently reflected in our metabolomic findings. Furthermore, the presence of numerous predicted secondary metabolite gene clusters suggested a latent capacity for producing bioactive compounds, potentially enhancing bioconversion to value-added products beyond basic degradation. Comparative genomic analysis with *I. lacteus* Irplac1 revealed high synteny (66.28% shared genes with 92.28% amino acid sequence identity), establishing *I. lacteus* J2 as a robust model strain for fundamental fungal biomass degradation research within its genus. While sharing a common genetic backbone, subtle variations in its enzymatic machinery or regulatory elements might contribute to its unique metabolic profile, warranting further investigation. Functional annotations based on KEGG, GO, and KOG databases further highlighted broad gene involvement in core metabolic pathways, signaling, and cellular quality control, underscoring the metabolic versatility of *I. lacteus* J2 and its adaptability to the available lignocellulosic substrate.

Untargeted metabolomic profiling provided dynamic and granular insights into the metabolic landscape of *I. lacteus* J2 during corn straw fermentation. A broad metabolite spectrum was detected, dominated by key classes such as organic acids and derivatives, lipids and lipid-like molecules, and heterocyclic compounds (Figure 3A), indicating active metabolic reprogramming in response to the lignocellulosic substrate. The abundance of organic acids in both positive and negative ion modes implies their crucial role in fungal degradation processes, including environmental acidification and lignin solubilization, as well as serving as metabolic precursors [27]. Significant amounts of lipids and lipid-like molecules may play roles in signal transduction, trafficking, morphological changes, and cell division [28]. KEGG pathway enrichment (Figure 4A,B) further elucidated these activities, revealing significant enrichment in global and overview maps, amino acid metabolism, carbohydrate metabolism, and lipid metabolism. Crucially, pathways related to glycan biosynthesis and the biosynthesis of other secondary metabolites were also enriched in the negative ion mode, highlighting *I. lacteus* J2’s capacity for not only deconstruction but also biosynthesis of potentially valuable compounds. LIPID MAPS analysis (Figure 4C,D) provided a detailed lipidome, with positive ion mode primarily annotating fatty acyls, glycerolipids, and glycerophospholipids, while negative ion mode enriched acidic subclasses, including glycerophosphoethanolamines, fatty acids, polyketides, flavonoids, and phenolic lipids. This diverse lipid profile, particularly the presence of phenolic lipids and enrichment in secondary metabolite biosynthesis, suggested sophisticated lipid-mediated signaling, structural adaptations, and a unique bioconversion potential during degradation, potentially distinguishing J2 from other fungi.

Temporal profiling across 3, 9, and 15 days (Figure 5) revealed substantial metabolic shifts and numerous differentially abundant metabolites. The progressive increase in upregulated metabolites from day 3 to day 15 (e.g., 167 up and 107 down in 9d vs. 3d POS; 210 up and 166 down in 15d vs. 3d POS) indicated a highly dynamic and adaptive strategy for efficient corn straw utilization. This metabolic flexibility, characterized by the sequential activation of different metabolic pathways and the coordinated production of specific breakdown products over time, is critical for its success in complex natural environments and industrial applications. For instance, early-stage upregulation of carbohydrate-related metabolites would align with initial polysaccharide breakdown, while later-stage accumulation of aromatic compounds and secondary metabolites could signify lignin modification and valorization. Further, correlation analysis of these differential metabolites (Figure 6) provided critical insights into the underlying metabolic networks and co-regulatory mechanisms. Strong positive correlations between metabolites like nicotinamide, 4-methylumbelliferyl-β-D-xylopyranoside, Dynorphin B (10–13), Alopecuridine, and Amastatin (Figure 6A) suggested co-regulated pathways, potentially involving core metabolic processes and carbohydrate breakdown products. For example, 4-methylumbelliferyl-β-D-xylopyranoside, a xylanase substrate analog, is positively correlated with other metabolites, indicating its central role in the coordinated degradation of hemicellulose. Conversely, negative correlations (e.g., between nicotinamide and Piptocarphin D, 6-Hydroxy nicotinic acid, and Pseudo-anisatin in Figure 6A, and rishitin with Nε,Nε,Nε-trimethyllysine and hydroxypropyl-glutamate in Figure 6C) highlight competitive pathways or shifts in metabolic flux, revealing the fungus’s sophisticated resource allocation strategies. The intricate relationships within specific clusters, such as the coordinated activity of several amino acids and related compounds including kynurenic acid (Figure 6E), or the distinct positive correlations among 12,13-epoxy-9-hydroxy-10-octadecenoate, Tamarixetin, Tagitinin D, and 13-OxoODE (Figure 6D), illustrated *I. lacteus* J2’s sophisticated metabolic orchestration, where various biochemical processes are tightly interconnected to optimize resource utilization and bioconversion efficiency. These network insights are crucial for understanding the holistic metabolic response to lignocellulose.

By integrating high-quality genomic data with dynamic metabolomic profiles, including temporal changes and correlation networks, this study offers a holistic and unprecedented view of the lignocellulose degradation mechanisms of *I. lacteus* J2. Our findings significantly advance our understanding of the genetic basis and metabolic versatility enabling this white-rot fungus to efficiently degrade lignocellulose. The comprehensive multi-omics analysis revealed several unique features distinguishing *I. lacteus* J2 from other fungi: (1) a robust and diverse lignocellulolytic enzyme system encoded in its genome, particularly rich in CAZymes and oxidoreductases capable of broad substrate degradation; (2) a highly dynamic and adaptable metabolic profile, characterized by significant temporal shifts in metabolite abundance and specific enrichment in secondary metabolite biosynthesis pathways; (3) the capacity to produce a broad spectrum of value-added compounds, including specific lipids (e.g., phenolic lipids, flavonoids) and heterocyclic compounds, indicating a strong bioconversion potential beyond simple degradation; and (4) a finely tuned and interconnected metabolic network that orchestrates efficient bioconversion over time, as evidenced by metabolite correlation patterns.

## 5. Conclusions

This study employed an integrated multi-omics strategy, combining high-quality whole-genome sequencing with untargeted metabolomics to comprehensively decipher the molecular mechanisms underlying highly efficient lignocellulose degradation by *I. lacteus* J2 grown on corn straw. Genomic analysis revealed that *I. lacteus* J2 possessed a robust enzymatic machinery, including an extensive repertoire of CAZymes and oxidoreductases, which largely explain its remarkable lignocellulose degradation capabilities. Functional annotation highlighted a wealth of genes involved in diverse metabolic pathways, while comparative genomics confirmed strong evolutionary conservation. The identification of a broad spectrum of differentially accumulated metabolites, particularly those orchestrating amino acid, carbohydrate, lipid, and secondary metabolic pathways, provided comprehensive mechanistic insights into the fungal response and resource utilization during bioconversion. Temporal and correlation analyses further illuminated the coordinated metabolic activities driving this process. These findings profoundly advance our understanding of the intricate molecular strategies employed by white-rot fungi for efficient lignocellulose degradation. This work not only provides a solid foundation for targeted genetic engineering to further enhance the bioconversion potential of *I. lacteus* J2 but also paves the way for its industrial application in the sustainable valorization of agricultural residues.

## Figures and Tables

**Table 1:** PacBio read statistics, assembly statistics, and genome features of *I. lacteus* J2.

Feature	Value
Valid ZWM number	127,431
Subreads number	1,546,034
Subreads total bases (bp)	15,016,142,153
Subreads mean length (bp)	9712
Subreads N50 (bp)	10,169
Subreads N90 (bp)	6630
Subreads max length (bp)	245,281
Subreads min length (bp)	2000
Number of contigs	114
Total length (bp)	46,386,314
Max length (bp)	4,106,595
Min length (bp)	9353
N50 (bp)	2,420,162
N90 (bp)	297,826
GC Content (%)	50.22
CDS number	14,647
tRNAs	295
rRNAs	33
snRNAs	45
miRNAs	98

Note: Subreads Number, the number of subreads after filtering; Subreads total bases, data size of all subreads; tRNA: Transfer RNA; rRNA: ribosomal RNA; snRNA: small nuclear RNA; miRNA: microRNA.

**Table 2:** Lignocellulose degradation-related genes in *I. lacteus* J2.

Substrate	Gene ID	Function
Cellulose	GME1266_g	Endoglucanase
	GME11700_g	Exoglucanase/Cellobiohydrolase
	GME12463_g	β-glucosidase
Hemicellulose	GME2307_g	Xylanase
	GME5730_g	β-xylosidase
	GME10460_g	Mannanase
	GME10049_g	β-mannosidase
Lignin	GME3599_g	Laccase
	GME7340_g	Lignin peroxidase
	GME12823_g	Manganese peroxidase
	GME12823_g	Versatile peroxidase

## Data Availability

The data available were submitted to a publicly accessible repository. This whole-genome sequencing project has been deposited at DDBJ/ENA/GenBank under the accession numbers SUB15553731. The raw metabolomics data have been deposited in the MetaboLights database with the accession number MTBLS12890. The original contributions presented in this study are included in this article; further inquiries can be directed to the corresponding authors.

## Abbreviations

The following abbreviations are used in this manuscript:

NGS	Next-generation high-throughput sequencing
SMRT	Single-molecule real time
CCTCC	China Center for Type Culture Collection
PDA	potato dextrose agar
DNBs	DNA nanoballs
cPAS	Combinatorial probe-anchor synthesis
CCS	Circular Consensus Sequencing
GO	Gene Ontology
KEGG	Kyoto Encyclopedia of Genes and Genomes
COG	Clusters of Orthologous Groups
NR	Non-Redundant Protein Database database
TCDB	Transporter Classification Database
CAZY	Carbohydrate-Active enZYmes
UHPLC-MS/MS	Ultra High-Performance Liquid Chromatography-Tandem Mass Spectrometry
CV	Coefficient of variation
PLS-DA	Partial least squares discriminant analysis
PCA	Principal component analysis
tRNAs	Transfer RNAs
rRNAs	ribosomal RNAs
snRNAs	small nuclear RNAs
miRNAs	microRNAs
ZWMs	Zero-Mode Waveguide stands
XCMS	eXtracted Chromatographic Mass Spectrometry
LC-MS/MS	Liquid Chromatography—Tandem Mass Spectrometry
